# Compromised Defenses: Exploitation of Epithelial Responses During Viral-Bacterial Co-Infection of the Respiratory Tract

**DOI:** 10.1371/journal.ppat.1005797

**Published:** 2016-09-15

**Authors:** Jeffrey A. Melvin, Jennifer M. Bomberger

**Affiliations:** Department of Microbiology and Molecular Genetics, University of Pittsburgh, Pittsburgh, Pennsylvania, United States of America; The University of North Carolina at Chapel Hill, UNITED STATES

## Introduction

**Fig 1:**
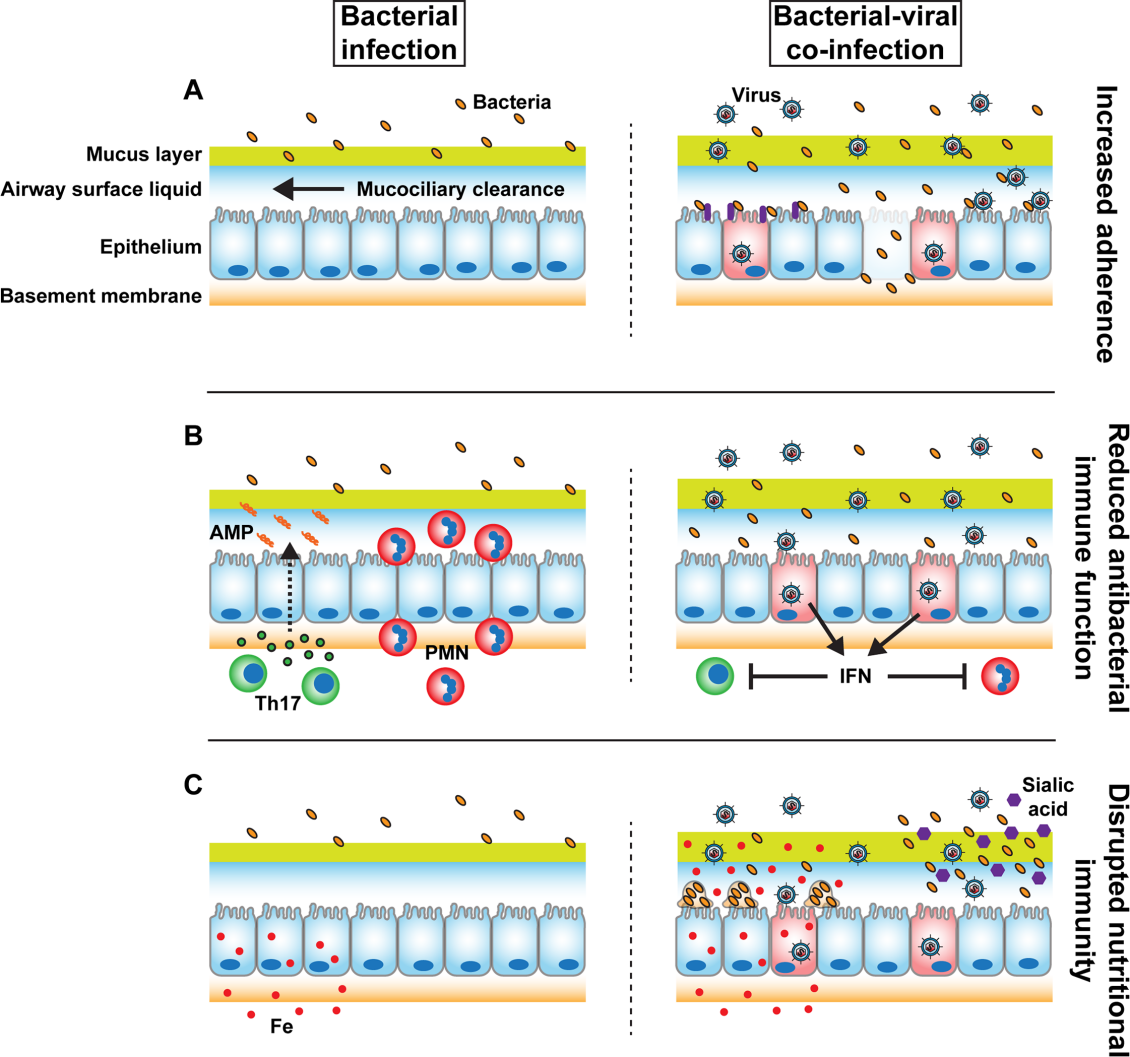
Model of increased susceptibility to secondary bacterial infection after primary viral infection of the respiratory epithelium. (A) The respiratory epithelium restricts bacterial attachment via mucociliary clearance and maintenance of cell–cell junctions, which restricts access to bacterial receptors. During viral infection, ciliary beat is reduced, barrier function is disrupted, bacterial receptors (purple) are upregulated, and direct viral–bacterial interactions lead to increased bacterial adherence to the epithelium. (B) The respiratory epithelium recruits and activates neutrophils or polymorphonuclear (PMN, red) cells and T helper cells (particularly Th17, green) in response to detection of bacterial infection or pro-inflammatory cytokines. These effects lead to influx of neutrophils and stimulation of epithelial antimicrobial peptide/protein (AMP, orange spirals) production in response to IL-17 receptor signaling (green circles, dashed arrow) by the epithelium. During viral infection, epithelial cells produce interferons (IFN), which skew the immune status towards antiviral activity, suppressing neutrophil, Th17 responses, and other antibacterial functions. (C) To inhibit microbial growth, the respiratory epithelium actively restricts nutrient availability in the airway lumen, including limitation of luminal iron concentrations (Fe, red circles). During viral infection, interferon production leads to dysfunctional iron limitation, stimulating biofilm biogenesis. In the case of influenza infection, influenza stimulates mucin secretion and cleaves sialic acid (purple hexagons) from secreted mucins via neuraminidase activity. Common upper respiratory tract commensal bacteria can utilize liberated sialic acid as a nutrient.

Respiratory infections are the greatest single contributor to the overall burden of disease worldwide [1]. Polymicrobial infections are becoming increasingly recognized in terms of both prevalence and their effect on disease severity, causing many common diseases such as oral infections, otitis media, chronic wound infections, and implanted medical device infections, as well as chronic pulmonary disease in cystic fibrosis patients [2,3]. There is a large body of literature demonstrating synergy between viral and bacterial infections at mucosal surfaces; for example, (i) the intestinal microbiota promotes enteric viral infection via direct interactions and modulation of the immune system [4]; (ii) sexually transmitted viruses exploit the altered environment, including altered pH, inflammatory, and oxidative settings, during bacterial vaginosis or aerobic vaginitis to increase infection of the vaginal and cervical epithelium [5]; and (iii) bacteria take advantage of the altered innate and adaptive immune responses of the respiratory tract during viral infection of the respiratory epithelium to increase infectivity and virulence [6]. Many studies have focused on the consequences of influenza infection on secondary bacterial infection in the respiratory tract, where altered lung physiology and immune status increases susceptibility to severe secondary bacterial infections with common commensal organisms of the upper respiratory tract, such as *Streptococcus pneumoniae* and *Staphylococcus aureus* [7]. Here, we focus on the role of the respiratory epithelium in defending against microbial pathogens as well as in facilitating synergistic pathogenic interactions (Fig 1).

## Role of the Respiratory Epithelium in Innate and Adaptive Immunity

The respiratory epithelium is the primary site of host–pathogen encounter in the respiratory tract and the first line of defense against infection (extensively reviewed [8–10]). The epithelium employs a wide array of tools to inhibit colonization as well as dictate innate and adaptive immune responses to pathogens that overcome these initial barriers. First and foremost, the epithelium is a physical barrier to pathogen invasion, forming cell–cell junctions to exclude pathogens from the underlying tissues and actively restrict nutrient availability. Respiratory epithelial cells maintain an airway surface liquid into which they secrete mucins that form a mucus layer to trap and propel pathogens out of the respiratory tract via mucociliary beat. The epithelium also secretes many additional effectors in the airway surface liquid, including antimicrobial peptides and proteins, such as degradative enzymes, iron sequestration proteins, protease inhibitors, collectin surfactant proteins, chemokines, and various forms of palate-lung-nasal-clone protein (PLUNC). The airway epithelium also produces toxic reactive nitrogen species and reactive oxygen species. These direct antimicrobial effectors are upregulated in response to pathogen or pro-inflammatory signals. Airway epithelial cells also produce cytokines and chemokines in response to pathogens, via pattern recognition receptor (PRR) activation, or to pro-inflammatory signals to shape the innate and adaptive immune responses, leading to an influx of neutrophils and monocytes, differentiation of monocytes into dendritic cells and macrophages, influx and activation of Th cells, and differentiation of B cells. B cell differentiation leads to immunoglobulin (Ig) class switching, and epithelial cells transcytose polymeric IgM and IgA into the airway surface liquid. Despite this array of defense functions, synergistic interactions between viral and bacterial respiratory pathogens can arise when one pathogen is able to suppress the antimicrobial activities of the epithelium or when an epithelial response that is protective against one pathogen makes the airway more permissive for infection by the other pathogen.

## Viral Infection Increases Bacterial Adherence to the Respiratory Epithelium

Viral infection of respiratory epithelium can increase bacterial adherence and colonization (as well as viral adherence) through multiple distinct mechanisms (Fig 1A). Through viral infection-induced damage of the respiratory epithelium, basal cells and basement membranes become exposed. Bacteria can more readily adhere to these underlying surfaces than to healthy ciliated epithelium [11,12]. The cell surface presentation of bacterial receptors, such as integrins, on airway epithelial cells can be upregulated by viral infections via induction of acute phase pro-inflammatory cytokines or TGF-β, leading to increased bacterial adherence to and colonization of the respiratory tract [13,14]. Viruses can also directly bind to bacteria and mediate more intimate interactions with the respiratory epithelium [15–17]. In the case of respiratory syncytial virus (RSV) and *S*. *pneumoniae*, the RSV G protein binds to penicillin binding protein 1a of *S*. *pneumoniae*, leading to increased adherence to ciliated epithelium, increased expression of *S*. *pneumoniae* virulence factors, and increased virulence [18]. Additionally, viral infection can indirectly increase bacterial adherence and colonization by decreasing mucociliary velocity [19]. The magnitude of these effects can vary depending on the strain of virus, strain of bacteria, and epithelial cell type [11,13], and a combination of these effects is likely responsible for the increased adherence observed in vivo.

## Viral Infection Skews the Antibacterial Immune Function at the Respiratory Epithelium

Respiratory viral infection skews the immune status of the respiratory tract to predispose to secondary bacterial infection (Fig 1B), which has been extensively studied for influenza [20]. The epithelium initiates this phenomenon by production of type I and III interferons in response to PRR-mediated detection of viral infection [21], which has important antiviral effects while leading to diminished antibacterial activity of the epithelium. Type I interferons, in response to influenza A infection, block production of phagocyte chemoattractants by the respiratory epithelium [22], impairing antibacterial neutrophilic responses. Type I interferon signaling, in response to influenza A infection, also inhibits stimulation of Th17 and downstream cytokine-mediated signaling [23], blocking antimicrobial peptide production by the respiratory epithelium [24,25]. Type III interferons are also detrimental to defense against Gram-positive and Gram-negative bacterial infection [26], though the precise mechanisms involved during viral–bacterial co-infection remain to be determined. Recent studies indicate that Type III interferon signaling induces changes in barrier function and microbiome shifts [27], as well as breakdown in nutritional immunity [28] that support secondary bacterial infections.

Secondary viral infections are also subject to modulation by prior bacterial infection in the respiratory tract. Bacterial infection can reduce virulence during subsequent viral infection, either via induction of an anti-inflammatory M2 phenotype in alveolar macrophages [29] or upregulation of antiviral interferon-stimulated genes (ISG) in epithelial cells [30]. However, development of protective immunity can be compromised, as *S*. *pneumoniae* infection can inhibit proper B cell maturation and production of protective antibodies during secondary influenza infection [31]. *Pseudomonas aeruginosa* can also disrupt antigen presentation in the respiratory epithelium needed for induction of an effective adaptive immune response [32], compromising viral clearance mechanisms and leaving the host more susceptible to subsequent viral infections. Secondary viral infection can also induce biofilm dispersal and bacterial dissemination in established bacterial infections [33,34], potentially contributing to the observation that transmission of colonizing *S*. *pneumoniae* is increased after influenza infection [35]. The mechanisms underlying these phenomena largely remain to be defined and likely require an intermediary role of the respiratory epithelium.

## Bacteria Take Advantage of Disrupted Nutritional Immunity during Respiratory Viral Infection

Nutritional immunity postulates that due to the necessity of trace metals for microbial growth, respiration, and metabolism, the host employs many regulatory pathways to sequester these nutrients [36]. Iron is one such nutrient that is crucial for host and microbial cell function alike, and, thus, it is tightly regulated in the host through complex interactions between uptake, storage, and use in the cell. While iron is well characterized for its importance in bacterial pathogenesis, dysregulation of iron homeostasis during viral infections and its role in co-infection are not well understood. We have recently demonstrated that iron levels are increased in the airway surface liquid during respiratory syncytial virus infection, in the form of transferrin-bound iron [28]. Moreover, this increase in iron availability is required for virus-induced bacterial biofilm growth by *P*. *aeruginosa* (Fig 1C). Consistent with altered iron homeostasis during respiratory viral infections, lipocalin-2 levels are altered in the airway during influenza A infection, and exogenous delivery of lipocalin-2 rescues viral exacerbation of *S*. *aureus* infection by reducing iron levels in the airway lumen [25]. As has been proposed for treatment of chronic bacterial infections [37], these studies suggest that iron chelation therapy might be worthy of consideration in conjunction with antimicrobials during viral–bacterial co-infections.

While nutritional immunity is typically defined as the restriction of trace minerals by the host to limit pathogenicity during an infection, we propose that this nutrient limitation also includes amino acids and other host-derived nutrients. The amino acid tryptophan is required for *Toxoplasma gondii* and *Chlamydia trachomonis* infections, and limitation of microbial access by the ISG indoleamine 2,3-dioxygenase (IDO1; [38,39]) is an important host defense mechanism to control infection. Also, the saccharide sialic acid liberated from host glycoconjugates has been demonstrated to fuel bacterial superinfection (Fig 1C). A recent study by Siegel and colleagues demonstrates that upregulation of the mucin MUC5AC during influenza A infection is used as a nutrient source for sialic acid, after liberation by viral and *S*. *pneumoniae* neuraminidases, and facilitates bacterial replication in the airway [40]. Additional studies defining the mechanisms by which viral effectors or host antiviral defenses disrupt nutritional immunity mechanisms to enable secondary bacterial infections may uncover new therapeutic targets for treatment of co-infections.

## Viral–Bacterial Co-Infection Antimicrobial Susceptibility and Potential Therapy

Polymicrobial infections can reduce antimicrobial susceptibility and complicate treatment [41]. For example, viral infection of the respiratory tract is correlated with treatment failure for otitis media [42,43]. Furthermore, respiratory viral infection can stimulate biofilm biogenesis by co-infecting bacterial pathogens, resulting in decreased susceptibility to frontline antibiotics [44]. There has therefore been a recent push to use antiviral therapy to reduce secondary bacterial infection, with some success [45]. Interestingly, neuraminidase inhibitors that can inhibit both influenza and streptococcal neuraminidases were able to block synergy for influenza A pathogenesis in vitro [46], an approach that may also prove effective for the bacterial infection, considering the role of neuraminidases in facilitating secondary *S*. *pneumoniae* infection [40]. As a broader approach, it was recently demonstrated that engineered antimicrobial peptides are simultaneously effective against both the enveloped virus RSV and recalcitrant *P*. *aeruginosa* biofilms [44], suggesting antimicrobials can be developed that have dual efficacy against both pathogens in a viral–bacterial co-infection. Due to the complexity of polymicrobial infections, complicated by the intermediary role of the host, antimicrobials capable of inhibiting both bacterial and viral pathogens simultaneously may be the most effective therapeutic approach and should be explored to a greater extent in the future.
